# *Odocoileus virginianus PRNP* sequencing reveals AF (Q_95_G_96_/H_95_G_96_) advantage over AC (Q_95_G_96_/Q_95_S_96_) against chronic wasting disease

**DOI:** 10.1186/s13567-026-01752-8

**Published:** 2026-05-26

**Authors:** Evan W. London, Alfred L. Roca, Yasuko Ishida, Tooba Latif, Jan E. Novakofski, Nohra E. Mateus-Pinilla

**Affiliations:** 1 Illinois Natural History Survey | Prairie Research Institute, University of Illinois Urbana-Champaign, 1816 S. Oak Street, Champaign, IL 61820 USA; 2 Department of Animal Sciences, University of Illinois Urbana-Champaign, 1207 W. Gregory Drive, Urbana, IL 61801 USA; 3 Carl R. Woese Institute for Genomic Biology, University of Illinois Urbana-Champaign, 1206 W. Gregory Drive, Urbana, IL 61801 USA; 4 Department of Pathobiology, University of Illinois Urbana-Champaign, 2001 S. Lincoln Avenue, Urbana, IL 61801 USA; 5 Department of Natural Resources and Environmental Sciences, University of Illinois Urbana-Champaign, 1102 S. Goodwin Avenue, Urbana, IL 61801 USA

**Keywords:** Cervid, transmissible spongiform encephalopathy, wildlife epidemiology, deer, CWD management, CWD surveillance

## Abstract

Chronic wasting disease (CWD) is a highly transmissible spongiform encephalopathy impacting cervids. Variations in the prion protein gene (*PRNP*), encoding the prion protein (PrP), influence disease progression and susceptibility. Protein sequence is a key determinant of PrP strains and their capacity for interspecies transmission. Sequencing *PRNP* in white-tailed deer has revealed non-synonymous polymorphisms impacting disease—c.285A > C (Q95H) and c.286G > A (G96S). Previous *PRNP* amplification protocols misclassified homozygous and heterozygous deer due to allelic dropout by a widely used forward primer. We resequenced 586 deer previously classified as homozygous using a redesigned primer (designated Ov-*PRNP*-F2) and found 128 heterozygotes. Subsequently, we sequenced additional CWD-positive and CWD-negative deer using Ov-*PRNP*-F2 to correct for dropout and then estimated PrP variant susceptibility and CWD risk using 778 CWD-positive and 3,070 CWD-negative deer from 22 Illinois counties, the largest *PRNP* sequence dataset to date. Animals encoding PrP variant combination C (96S) and F (95H) were the least susceptible to CWD (OR = 0.03, *p* < 0.001) compared to deer encoding two copies of PrP variant A 95Q;96G. A novel finding is that deer encoding a single copy of PrP 95H were less susceptible to CWD compared to those with a single copy of PrP 96S (OR = 0.40, *p* < 0.001). The results support using genotyping methods to map susceptibility to CWD across the landscape, focusing on PrP variant frequencies to improve CWD epidemiological risk models.

**Supplementary Information:**

The online version contains supplementary material available at 10.1186/s13567-026-01752-8.

## Introduction

Chronic wasting disease (CWD) is a highly transmissible spongiform encephalopathy (TSE) of cervids [1]. Like all prion diseases, CWD manifests through the conversion of the cellular prion protein (PrP^C^) into the misfolded infectious prion protein isoform (PrP^Sc^) [2, 3]. Infection can be through direct contact with prions shed by positive animals or indirectly through the environment, where prions can remain infectious for extended periods [4]. The prion protein gene encodes PrP^C^ [5] and variations in the PrP amino acid sequence determine tropism during prion infection [3]. Additionally, the PrP sequence is determinant of strain emergence and capacity for interspecies transmission [6, 7]. While initially detected in mule deer (*Odocoileus hemionus*) in Colorado in 1967, CWD has been found in several cervid species, including white-tailed deer (*Odocoileus virginianus*), wapiti (elk, *Cervus canadensis*), moose (*Alces alces*), and red deer (*Cervus elaphus*). As of publication, the prevalence of CWD is increasing, and the disease has spread to free-ranging cervids in 36 US states and 4 Canadian provinces in North America and has also been detected in reindeer (*Rangifer tarandus*) in the Arctic (Norway, Finland, and Sweden) [8, 9]. Although there are species differences between PrP^C^ sequences, the greater amino acid identity shared increases the likelihood of intra- and interspecies transmission [10, 11]. With the possibility of human transmission not ruled out, the spread of CWD has biosecurity implications and unknown potential to impact the food supply.

As prion diseases do not elicit an immune response, the genetics underlying PrP expression play a crucial role in disease susceptibility and progression. In this context, susceptibility refers to the individual's likelihood of being influenced or harmed by a particular pathogen. Animals may express two different PrP variants when non-synonymous *PRNP* alleles are present. Therefore, characterizing PrP variation is key to managing TSEs in wildlife. White-tailed deer (*Odocoileus virginianus*; WTD) has high population densities and a widespread distribution, making it one of the species most impacted by CWD [12]. Additionally, species that predate upon or scavenge carcasses of CWD-positive WTD (e.g., wild pigs) may be at risk for interspecies transmission [13]. Recent detection of subclinical prion infection in wild pigs (*Sus scrofa*) is of particular concern [13], considering unknown impacts of newly adapted strains [14, 15].

**Figure 1:**
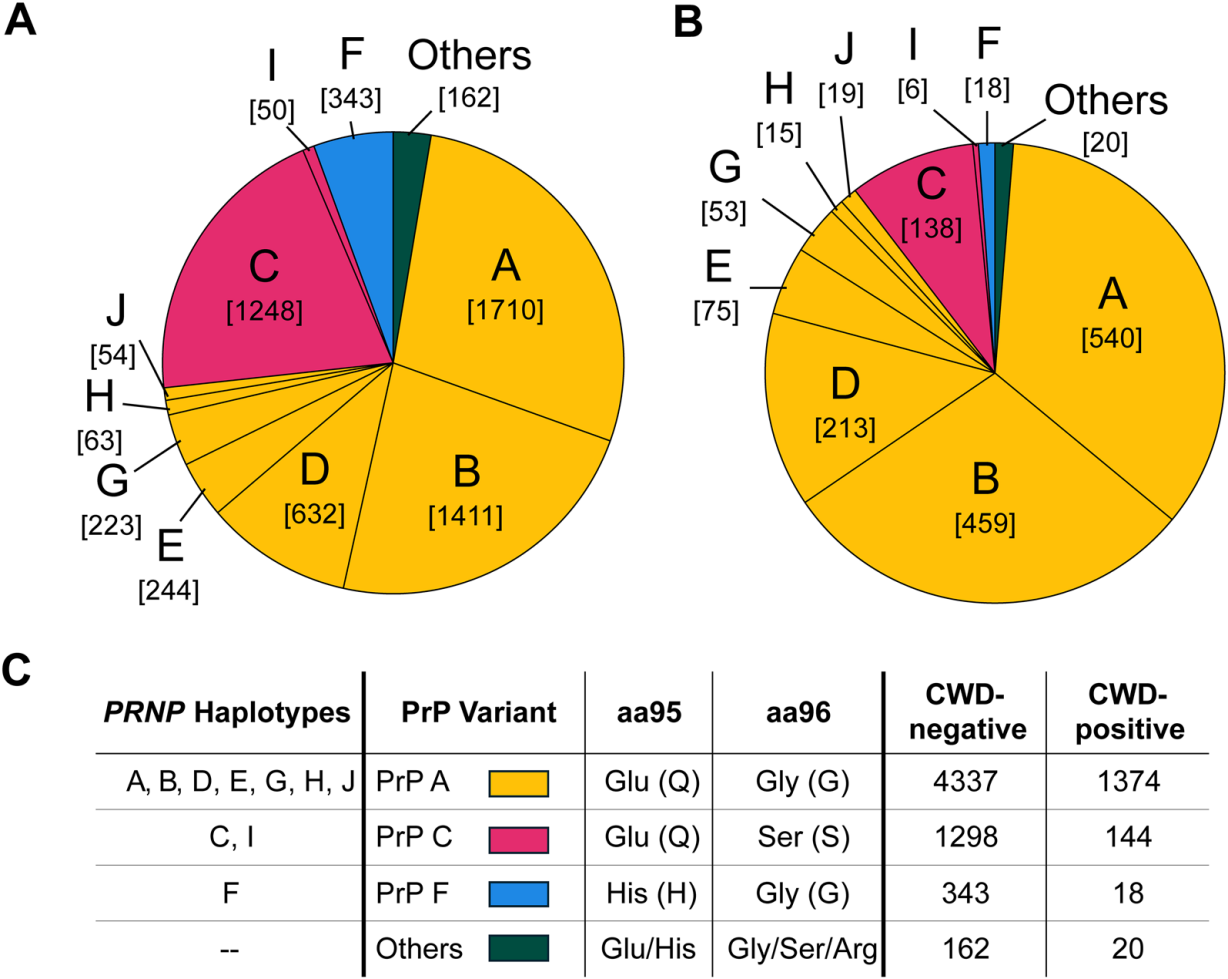
***PRNP*** **haplotypes revealing an uneven distribution between groups of CWD-positive and CWD-negative deer.** Frequencies of *PRNP* haplotypes are shown as pie charts for both **A** CWD-negative and **B** CWD-positive groups. Synonymous haplotypes encoding PrP variant A are represented in mustard (A, B, D, E, G, H, J), variant C in pink (C, I), and variant F in blue. The number of chromosomes encoding each haplotype is represented in brackets below the haplotype labels. The table **C** shows the total number of *PRNP* haplotypes encoding the PrP variants. Haplotypes with a population frequency below 0.5% are grouped as “Others” in dark green (*n* = 29). Haplotypes C, F, and I were more prevalent in CWD-negative deer than in CWD-positive deer. The amino acid identities at PrP positions 95 (aa95) and 96 (aa96) are shown for the major PrP variants A, C, and F. “Other” haplotypes encode multiple PrP variants with substitutions. Less frequent haplotypes (dark green) were also more common in CWD-negative deer. In contrast, haplotypes encoding variant A were found in a lower proportion of CWD-negative deer compared to CWD-positive deer.

Multiple single-nucleotide polymorphisms (SNPs) or genetic mutations have been identified within the 771 bp coding region of WTD *PRNP* gene, indicating a change in the DNA sequence and designated with alphanumerically labeled haplotypes (shown in Fig. 1) [16–18]*.* The DNA sequence of the *PRNP* gene determines the chain of amino acids in the PrP protein variant expressed in the host. Two non-synonymous SNPs in the *PRNP* coding region, c.285A > C (coding base pair 285, Adenine [A] replaced by Cytosine [C]) and c.286G > A (coding base pair 286, Guanine [G] replaced by Adenine [A]), result in amino acid changes Q95H and G96S in the 256 amino acid PrP protein and have been associated with an advantage against CWD [18–22]. Both PrP amino acid residues 95 and 96 are located near the sequence boundary between the octapeptide repeat region (aa63-aa86) and the first beta sheet, which has implications for PrP^Sc^ misfolding propensity and biological relevance to CWD susceptibility [23, 24]. Associations with slower disease progression were shown for PrP variants F (95H;96G) and C (95Q;96S) in animals orally infected with CWD [25], and two animals with a Histidine (H) at codon 95 had the longest incubation periods and the longest survival, indicating that the Q95H substitution has the greatest effect. In previous studies of Illinois WTD, animals expressing PrP variants C or F were significantly less associated with CWD than animals exclusively expressing the most common variant, PrP A (95Q;96G) [19–21]. However, previous CWD risk analyses did not find a statistical difference between animals expressing a combination of PrP A and C and the combination of PrP A and F variants, and it remained unclear which protein variant combination is more advantageous against CWD [20].

Previous sequencing efforts were based on PCR amplification of WTD *PRNP* using a primer pair developed in 2004, CWD-223 (forward)/CWD-224 (reverse) [22]. A major limitation of this primer pair is that it can fail to amplify chromosome copies of *PRNP* due to variation at the binding site of primer CWD-223 [26]. This primer-associated allelic dropout restricts the identification of animal genotypes. A new pair of primers, WTDPRNP-F/WTDPRNP-R, was developed by Haley et al. to avoid allelic dropout [26]. Deer classified as homozygous in Illinois by primer pair CWD-223/CWD-224 were resequenced using primer pair WTDPRNP-F/WTDPRNP-R, and 18% were found to be heterozygous [27].

While the primers presented by Haley et al. successfully amplified *PRNP*, Sanger sequencing was “performed unidirectionally using reverse primer [CWD] 224”. However, we sought to use a forward primer with an amplification protocol compatible with CWD-224 that maximizes the efficiency of laboratory protocols and retains the ability to detect allelic dropout. Studies of *PRNP* frequencies in Illinois WTD have used the primer pair developed by O’Rourke et al., leading to imprecise frequency estimates [22]. Evidence of allelic dropout in *PRNP* sequencing [27] suggests that previous *PRNP* genotype frequencies in Illinois WTD might be underestimated. Because herd genetics can impact disease spread across the landscape, it is important to obtain accurate frequency estimates of advantageous haplotypes influencing the risk of CWD. Therefore, we aimed to 1) utilize and validate an updated protocol free of allelic dropout for accurate evaluation of *PRNP* haplotype frequencies, 2) determine PrP variants associated with allelic dropout through resequencing of animals previously classified as homozygous, and 3) make refined estimates of protection against CWD by the PrP variants in the population using an expanded sample size and an updated primer set. Our findings show that the PrP variant F (95H) conferred the most advantage against CWD in WTD.

## Materials and methods

### Sample selection

Through the ongoing Illinois Department of Natural Resources CWD surveillance and management program, muscle tissue samples were sourced from 22 Illinois counties through hunter harvest, localized focused culling, and special permits for deer population control, nuisance removal, and scientific programs [28]. The sample selection strategy matched tissues from deer positive for CWD and two CWD-negative deer based on year and location. Based on the selection criteria, tissue from 414 positive and 926 negative deer were available for DNA extraction. Additional sequences of heterozygous deer (*n* = 2150; 1838 from [19, 21] and 312 from [20]) were included from previous *PRNP* studies. For homozygous samples from prior efforts (*n* = 772) [19–21], 586 with available archived tissue were selected for resequencing to evaluate the occurrence of allelic dropout [22, 26, 27]. All samples and references from their sources are detailed in Additional file 1.

### Primer validation

We utilized the primer Ov-*PRNP-*F2 [29] in our protocol to replace the forward primer CWD-223. The primer, Ov-*PRNP*-F2 (5'-ATGGGCATATGATGCTGACA-3’), was designed using Primer3 [30] to mitigate allelic dropout bind 17 bp upstream of the forward primer, CWD-223 [20]. Balanced GC content was maintained to give a melting temperature compatible with the reverse primer, CWD-224 (5'-AGAAGATAATGAAAACAGGAAG-3') [22]. To confirm haplotype identification, 36 deer with heterozygous genotypes determined by the CWD-223/CWD-224 primer pair [22] were re-sequenced with the new primer pair, Ov-*PRNP*-F2/CWD-224.

### DNA extraction, PCR amplification, and Sanger sequencing

Genomic DNA was extracted using the DNeasy Blood and Tissue kit (Qiagen Inc., Valencia, CA, USA) following the manufacturer's instructions. The complete *PRNP* coding region was amplified with the forward primer, Ov-*PRNP*-F2, and the reverse primer CWD-224. The PCR reaction included 0.5 μL of DNA (approx. 10 ng), 11μL of Nuclease-free water, 0.5 μL of each 20 μM primer Ov-*PRNP-*F2 and CWD-224, and 12.5 μL of 2 × GoTaq Green Master mix (Promega, WI, USA) for a total reaction volume of 25 μL. Conditions for PCR involved an initial denaturation at 95 °C for 5 min, followed by 36 cycles of 95 °C for 1 min, 55 °C for 1 min, 72 °C for 1 min, with a final extension of 72 °C for 5 min. Confirmation of successful PCR amplification was visualized using gel electrophoresis. Enzymatic purification of amplified PCR products used exonuclease I (New England Biolabs, USA, MA) and recombinant shrimp alkaline phosphate (New England Biolabs, USA, MA). Sanger sequencing reactions were completed following the protocol of ThermoFisher Scientific's Applied Biosystems BigDyeTerminator v3.1 cycle sequencing kit. In addition to PCR primers (Ov-*PRNP*-F2 and CWD-224), two internal primers, *PRNP*-IF (5'-ATGCTGGGAAGTGCCATGA-3'; 5’ at 394 bp) and *PRNP*-IR (5'-CATGGCACTTCCCAGCAT-3'; 5’ at 411 bp) [20, 27], were included to produce high-quality base calls in both forward and reverse directions along the whole length of the amplicon. Sequencing reaction products were submitted to the Roy J. Carver Biotechnology Center at the University of Illinois Urbana-Champaign for clean-up and capillary electrophoresis.

### *PRNP* sequence analysis and Bayesian haplotype inference

Capillary electrophoresis sequencing results from the Roy J. Carver Biotechnology Center were downloaded as ‘.abi’ files. All four sequencing reads (forward, reverse, internal-forward, internal-reverse) for each sample were trimmed at both ends and assembled into contiguous consensus sequences using the default settings of Sequencher (Gene Codes). Contigs were manually inspected for ambiguous base calls. Gaps and indels arising from sequencing errors were removed. All consensus sequences were aligned using MUSCLE [31] and trimmed to the 625 base pairs of the 771 bp *PRNP* coding region (aa20-aa228) that span variable sites previously identified in Illinois and Wisconsin (Additional file 2) [19–21, 27, 32]—sequences of 4076 deer served as a population sample for Bayesian haplotype inference. Phased sequences were produced using Bayesian inference by the PHASE algorithm [33, 34] implemented in DNASP6 [35]. Phased haplotype DNA sequences were translated into protein sequences using MEGA [36]. Phased nucleotide and protein sequences were input to a custom R script (Additional file 3) for matching phased sequences using the rBLAST package [37] and BLAST databases [38] of previously identified *PRNP* haplotypes and PrP variants (variants and their encoding haplotypes are shown in Additional files 4 and 5) [17, 19–21, 32].

### Estimation of protection against CWD conferred by PrP variants

Odds ratios were calculated using the PrP protein variants and immunohistochemistry test results of 3848 Illinois deer (3070 CWD-negative, 778 CWD-positive). Comparison groups were formed to calculate the odds ratios of the associations of PrP variants and animal-level protein variant combinations with CWD. PrP variant-level odds ratios were calculated between the three major PrP variants (>1% population frequency; A, C, and F) based on the CWD status of the deer bearing the chromosome that encoded the protein variant (Additional file 6).

Animal-level odds ratios were calculated using deer with six PrP variant combinations (A/A, A/C, A/F, C/C, C/F, F/F) and the CWD status of the animal. Deer encoding two copies of PrP variant A (A/A) served as the reference group most susceptible to CWD. Further comparisons used deer with semi-susceptible PrP variant combinations, PrP A/C and A/F. Fisher’s exact test was conducted using the R statistical package (Version 4.2.3) to account for the few deer in small genotype by CWD status categories (e.g., a single CWD-positive C/F deer) with effect sizes reported as odds ratios (OR) (Additional file 7) [39]. A two-sided alternative hypothesis was used to account for reduced or increased odds ratios of testing positive for CWD. An alpha of 0.05 was used to construct 95% confidence intervals. To account for the false discovery rate of repeated statistical tests, *p*-values were adjusted using the Benjamini–Hochberg procedure [40, 41]. All *p*-values from PrP variant and animal-level analyses were included in the procedure.

## Results

### Allelic dropout resolved by forward primer Ov-*PRNP*-F2

Allelic dropout by primer CWD-223 was predominant for haplotypes with the c.286G > A (96S) polymorphism. Through primer validation and the resequencing of 586 Illinois deer previously identified as homozygous using primer CWD-223, we updated genotype information for 128 animals as heterozygous using primer Ov-*PRNP*-F2 [19–21, 32]. To validate that Ov-*PRNP*-F2 will not cause allelic dropouts, consistency between primer CWD-223 and Ov-*PRNP*-F2 was examined in 36 heterozygous deer that were amplified with both primers (complemented with reverse primer CWD-224), and the *PRNP* genotype did not change. Of the 2736 deer tested for CWD and genotyped for *PRNP* in Ishida et al., 4.6% deer (128/2736) were identified as heterozygotes due to the identification of allelic dropout [20]. There was no significant difference (Fisher’s exact test, two-sided) in the frequency of allelic dropout by CWD status (4.8% CWD-negative: 3.4%, CWD-positive: *p* = 0.2). The highest dropout occurrences across the sequenced population were for haplotype C (2%, [56/2,736]) (Table 1). Notably, *PRNP* haplotype C had 56 dropout occurrences out of 967 haplotype copies previously undetected or without dropout (Table 1).

**Table 1:** **Allelic dropout underestimates frequencies of *PRNP*** **haplotypes C, I, and Odvi27, encoding 96S**

*PRNP*	Dropout Occurrences			Haplotype Copies (with dropout)	Haplotype Copies (without dropout)	Dropout frequency per-haplotype	PrP aa95	PrP aa96
	CWD- negative	CWD- positive	Total*					
A	18	1	19	1690	1662	0.011	Q	G
C	50	6	56	926	967	0.058	–	S
D	14	6	20	578	589	0.009	–	–
B	13	1	14	1393	1367	0.010	–	–
I	9	0	9	26	33	0.272	–	S
F	3	0	3	283	282	0.010	H	–
E	2	0	2	211	210	0.009	–	–
G	1	0	1	201	199	0.005	–	–
J	1	0	1	38	39	0.025	–	–
Odvi27	1	0	1	1	2	0.500	–	S
W	1	0	1	1	2	0.500	–	–
X	1	0	1	1	2	0.500	–	–
Total	114	14	128	–	–	–		

^*^Haplotypes ordered by total dropout occurrences, with haplotype A shown at the top for amino acid reference.

Primer-linked allelic dropout was primarily associated with haplotype C (56/128). Amino acids are reported for PrP residues 95 and 96, with a dash indicating no substitution from PrP A. Haplotype I, which encodes PrP C (96S), exhibited the highest per-haplotype dropout frequency (9 out of 33 haplotype copies). Allelic dropout was identified with primer Ov-*PRNP*-F2 but initially missed by primer CWD-223. Primer error frequencies were calculated per *PRNP* haplotype based upon the previously sequenced population of 2736 deer with CWD test results from Ishida et al. [20]. A subset of homozygous deer was re-sequenced and 21.8% (128/586) were determined to be heterozygous.

Overall, the haplotypes of 66 deer (C: 56; I: 9; Odvi-27: 1) in the population encoding PrP variant C were previously undetected. For *PRNP* haplotype I, which also encodes PrP variant C, dropout was identified in 0.3% (9/2,736) of the deer population. However, *PRNP* haplotype I had the highest per-haplotype dropout frequency (27%, 9 occurrences out of 33 copies) of haplotypes with > 0.5% population frequency (Table 1).

### Bayesian haplotype inference of *PRNP* sequences from 22 Illinois counties

We characterized *PRNP* genotypes for 4076 deer, an increase from 2899 animals by previous efforts [19–21], across 22 northern Illinois counties. Ten haplotypes occurred at greater than 0.5% population frequency based on the Bayesian phasing inference of 4076 aligned consensus sequences (625 bp of the *PRNP* coding region), yielding 8152 phased haplotypes, defined by 16 variable positions and representing 38 unique haplotypes (Additional file 2). Haplotypes above 0.5% population frequency were distributed as follows: A (29.1%, [2372/8152]), B (24.3%, [1980/8152]), C (17.8% [1458/8152]), D (11.0%, [895/8152]), E (4.3%, [350/8152]), F (4.7%, [383/8152]), G (3.6%, [292/8152]), H (1.0% [85/8152]), I (0.7% [63/8152]) and J (0.9%, [77/8152]) (Additional file 2, Additional file 8). Haplotype diversity ($Hd$) was 0.81, and nucleotide diversity ($\pi$) was 0.002 for the sample population. One novel haplotype to Illinois, OVC1, had previously been reported in Arkansas white-tailed deer and Florida key deer (*O. v. clavium*) [17, 42]. The median-joining network (MJN) had no inferred nodes [43] (Additional file 8). Translated haplotype sequences encoded five variable amino acid sites across 11 PrP variants (Additional file 4). Three PrP variants occurred at greater than 1% population frequency: A (74.9%, [6106/8152]), C (19.0%, [1549/8152]), and F (4.7%, [389/8152]) (Additional files 4 and 8).

### *PRNP* haplotype and PrP variant frequency by CWD status

Haplotypes were unevenly distributed across CWD-positive and CWD-negative deer (Figure 1). Of the deer genotyped for *PRNP,* CWD test status was available for 3848 Illinois deer (3070 CWD-negative, 778 CWD-positive) (Additional files 5 and 9), an increase from 2754 animals in previous datasets [19–21]. Chromosomes bearing haplotypes C and I (encoding variant C) were more common in CWD-negative deer (C: 20.3% [1248/6140], I: 0.8% [50/6140]) compared to CWD-positive deer (C: 8.8% [138/1556], I: 0.3% [6/1556]).

Similarly, Haplotype F (encoding variant F) was also more common amongst the chromosomes of CWD-negative deer (5.6% [343/6140] CWD-negative, 1.2% [18/1556] CWD-positive). Haplotype L (encoding PrP variant L A123T), occurring in a very small subset of the population chromosomes (39/7696), nominally followed the same pattern (CWD-negative, 0.6% [37/6140]; CWD-positive, 0.1% [2/1556]) (Additional file 9).

### Statistical associations between positive CWD cases and PrP variants

**Figure 2:**
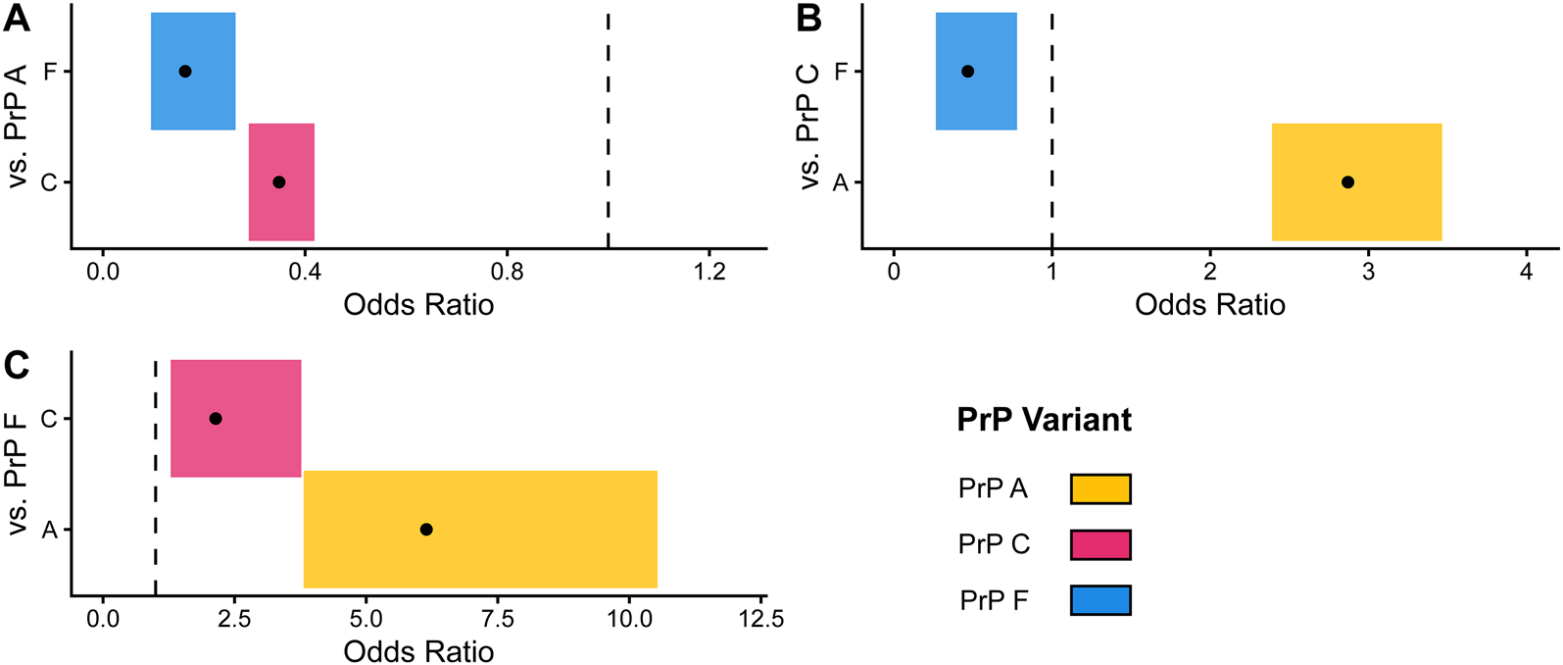
**Comparisons of prion protein variants reveal PrP F as more advantageous against CWD than PrP C**. Graph **A** PrP variants C (OR = 0.35, *p* < 0.001) and F (OR = 0.16, *p* < 0.001) were associated with significantly lower odds of CWD compared to PrP variant A. Graph **B** Furthermore, variant A showed significantly increased odds of CWD (OR = 2.87, *p* < 0.001) compared to variant C, while variant F had significantly lower odds than C (OR = 0.47, *p* = 0.002). Graph **C** PrP variants A (OR = 6.14, *p* < 0.001) and C (OR = 2.14, *p* = 0.002) had significantly higher odds of CWD compared to variant F. Reciprocal odds ratios are presented to facilitate visual comparisons between estimates. The point estimate of Fisher’s exact test is shown for all pairwise comparisons of PrP variants A (aa95Q, aa96G), C (aa95Q, aa96S), and F (aa95H, aa96G). Shaded areas represent the 95% confidence interval for the point estimate, and the dashed line represents the null hypothesis of the Fisher exact test (odds ratio [OR] = 1), equivalent to the odds ratio comparison of the variant against itself.

PrP variants C and F had significantly lower associations with CWD than variant A (C: OR = 0.35, *p* < 0.001, F: OR = 0.16, *p* < 0.001) (Fig. 2, Additional File 3), with PrP variant F also showing a significantly lower association with CWD than variant C (OR = 0.47, *p* = 0.002) (Figure 2, Additional file 6). Odds ratios were calculated for PrP variants present at > 1% frequency using the CWD test status of 7598 chromosomes encoding: A (4376 CWD-negative, 1387 CWD-positive), C (1322 CWD-negative, 146 CWD-positive), and F (349 CWD-negative, 18 CWD-positive) (Additional file 6).

### Associations between positive CWD cases and PrP variant combinations

**Figure 3:**
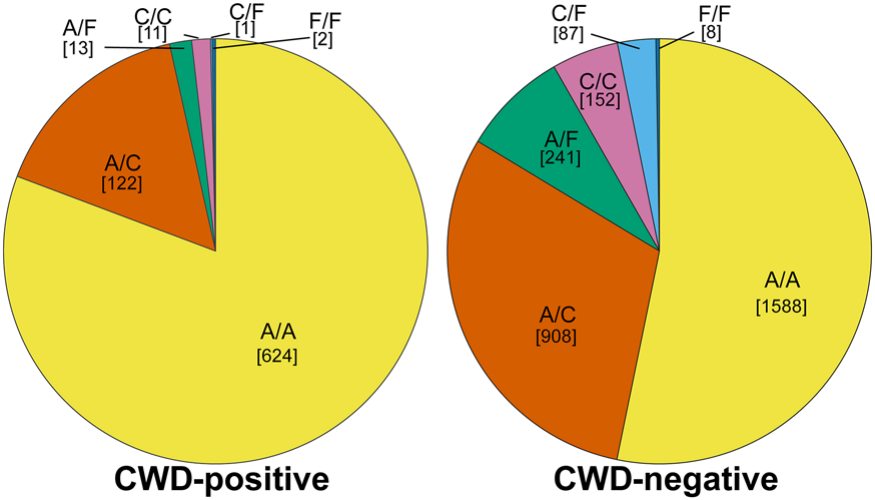
**Distribution of PrP variant combinations between CWD-positive and CWD-negative deer**. Pie charts of PrP variant combinations with greater than 1% population frequency. The number of deer corresponding to the PrP variant combination is noted in brackets. Only 1.8% (14/773) cases of CWD were found in animals encoding only PrP variants C or F (C/C, C/F, F/F), and just a single PrP variant combination C/F deer tested positive for CWD. In contrast, most cases of CWD, 80% (624/773), occurred in animals with PrP variant combination A/A, however, 16% (135/773) of cases occurred in animals with one copy of variant A(A/C, A/F)

**Figure 4:**
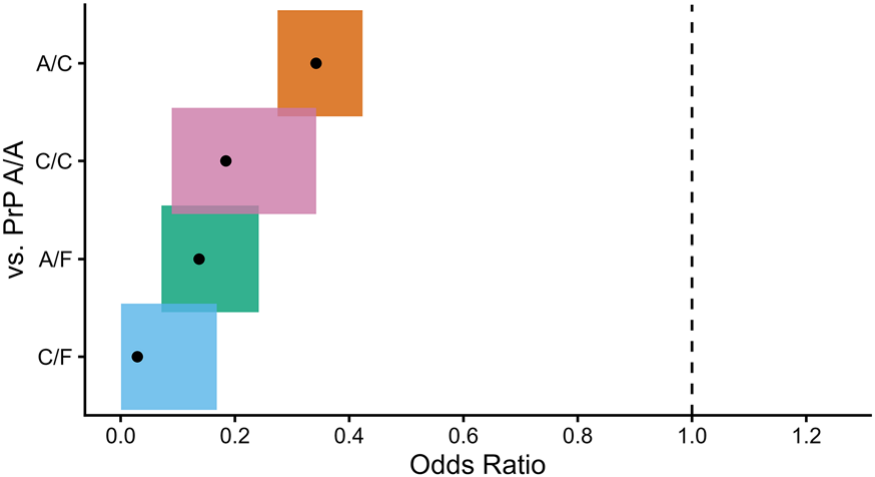
**Odds ratios of CWD status for deer with different PrP variant combinations compared against PrP combination A/A**: Deer with PrP combination C/F exhibit the lowest susceptibility to CWD. Black dots represent point estimates of odds ratios calculated using Fisher’s exact test. Shaded areas extending from each point estimate indicate 95% confidence intervals (α = 0.05). The dashed line represents the null hypothesis of Fisher’s exact test (odds ratio = 1) corresponding to the reciprocal comparison of PrP A/A deer. Odds ratios below 1 indicate reduced odds of testing CWD-positive compared with PrP A/A deer. The PrP variant combinations of comparison are labeled on the y-axis. The confidence intervals for odds ratios of A/F(OR=0.14, p < 0.001, [0.07 - 0.24]) and A/C (OR = 0.34, p < 0.001, [0.275 - 0.424]) did not overlap, indicating animals expressing both PrP variants A and F had lower CWD risk than animals expressing both PrP variants A and C. The protein variant combination with the most reduced odds of CWD was PrP C/F (OR = 0.03, p < 0.001); however, this estimate is based on a single C/F animal testing positive for CWD

Deer only expressing PrP variants C and F (C/C, C/F, and F/F) were significantly less susceptible to CWD (OR = 0.20, *p* < 0.001) compared to deer with PrP variant A (A/A, A/C, and A/F) (Table 2). Furthermore, deer expressing PrP A/F were less susceptible to CWD than deer expressing A/C (OR = 0.40, *p* < 0.001) (Table 2). Odds ratios were calculated at the animal level—considering the combination of variant copies, one for each chromosome—using the CWD test status of 3,757 deer expressing PrP variants present at > 1% frequency: PrP A/A (1,588 CWD-negative, 624 CWD-positive), PrP A/C (908 CWD-negative, 122 CWD-positive), PrP A/F (241 CWD-negative, 13 CWD-positive), PrP C/C (152 CWD-negative, 11 CWD-positive), PrP C/F (87 CWD-negative, 1 CWD-positive), PrP F/F (8 CWD-negative, 2 CWD-positive) (Figure 3). Relative to the deer with PrP variants A/A, the deer with PrP C/F were the least associated with CWD (OR = 0.03), followed by PrP A/F (OR = 0.14), C/C (OR = 0.18), and PrP A/C (OR = 0.34) (Figure 4 and Table 2). All animal-level odds ratios were statistically significant (*p* < 0.001) apart from PrP F/F (OR = 0.63, *p* = 0.734). (Table 2).

**Table 2:** **PrP C/F deer most protected against CWD vs. A/A, followed by A/F, C/C, and A/C.** Animal-level comparisons of single PrP variant combination groups with significantly reduced susceptibility relative to PrP A/A deer (rows 1-4) are visualized in Figure 4. Combined comparison groups were created based on possessing at least one encoded copy of PrP variant C (A/C, C/F, C/C), at least one encoded copy of variant F (A/F, C/F, F/F), and deer without variant A (C/C, C/F, F/F). Comparisons were also made considering deer that had at least one encoded copy of variant A against animals with the less susceptible variant C (A/A, A/C vs. C/F, C/C), F (A/A, A/F vs. C/F, F/F), or either variants C or F (A/A, A/C, A/F vs. C/C, C/F, F/F). All single protein variant combinations (A/A, C/C, F/F) were compared against all mixed variant combinations (A/C, A/F, C/F)

	Odds Ratio*	*p*-value	95% Confidence Interval	Group 1		Group 2	
Group 1 vs. Group 2				CWD negative	CWD positive	CWD negative	CWD positive
**A/A vs. C/F**	0.02	< 0.001	< 0.01–0.16	1588	624	87	1
**A/A vs. A/F**	0.13	< 0.001	0.07–0.24	1588	624	241	13
**A/A vs. C/C**	0.18	< 0.001	0.08–0.34	1588	624	152	11
**A/A vs. A/C**	0.34	< 0.001	0.27–0.42	1588	624	908	122
A/A, A/F vs. C/F, F/F	0.09	< 0.001	0.01–0.27	1829	637	95	3
A/A vs. A/F, C/F, F/F	0.12	< 0.001	0.06–0.20	1588	624	336	16
A/A vs. C/C, C/F, F/F	0.14	< 0.001	0.07–0.24	1588	624	247	14
C/C vs. C/F	0.15	0.061	< 0.01–1.13	152	11	87	1
A/A, A/C vs. C/F, C/C	0.16	< 0.001	0.08–0.30	2496	746	239	12
A/A, A/C, A/F vs C/C, C/F, F/F	0.20	< 0.001	0.10–0.35	2737	759	247	14
A/A vs. A/C, A/F	0.29	< 0.001	0.24–0.36	1588	624	1149	135
A/A vs. A/C, C/F, C/C	0.30	< 0.001	0.24–0.36	1588	624	1147	134
A/A, C/C, F/F vs A/C, A/F, C/F	0.30	< 0.001	0.24–0.36	1748	637	1236	136
A/C vs. A/F	0.40	< 0.001	0.20–0.72	905	122	241	13
A/C vs. C/C	0.53	0.060	0.25–1.02	905	122	152	11
A/A vs. F/F	0.63	0.734	0.06–3.20	1588	624	8	2

^*^Ordered by ascending odds ratio. Bold group comparisons visualized in Figure 4.

Pie charts of PrP variant combinations with greater than 1% population frequency. The number of deer corresponding to the PrP variant combination is noted in brackets. Only 1.8% (14/773) cases of CWD were found in animals encoding only PrP variants C or F (C/C, C/F, F/F), and just a single PrP variant combination C/F deer tested positive for CWD. In contrast, most cases of CWD, 80% (624/773), occurred in animals with PrP variant combination A/A, however, 16% (135/773) of cases occurred in animals with one copy of variant A (A/C, A/F).

Animal-level comparisons of single PrP variant combination groups with significantly reduced susceptibility relative to PrP A/A deer (rows 1-4) are visualized in Figure 4. Combined comparison groups were created based on possessing at least one encoded copy of PrP variant C (A/C, C/F, C/C), at least one encoded copy of variant F (A/F, C/F, F/F), and deer without variant A (C/C, C/F, F/F). Comparisons were also made considering deer that had at least one encoded copy of variant A against animals with the less susceptible variant C (A/A, A/C vs. C/F, C/C), F (A/A, A/F vs. C/F, F/F), or either variants C or F (A/A, A/C, A/F vs. C/C, C/F, F/F). All single protein variant combinations (A/A, C/C, F/F) were compared against all mixed variant combinations (A/C, A/F, C/F).

## Discussion

This study reports *PRNP* allele frequencies for the Illinois WTD population with improved accuracy and precision through a new amplification method and an increased sampling size. The expanded sampling of this study (2754 → 3848) identified significantly reduced susceptibility to CWD based on the PrP variants expressed by WTD. Our study finds WTD with the PrP variant F (95H) were significantly shown to be most protected against CWD when compared to PrP A or C, consistent with previous studies [25, 44, 45]. Identifying *PRNP* haplotype susceptibility can help focus wildlife management efforts in areas with high genetic susceptibility and update epidemiological risk models of CWD spread by incorporating genetic background to highlight areas at greater risk of disease.

We acknowledge the limitations inherent in studies with wild populations. First, we did not include live animals, since CWD testing uses post-mortem tissues, therefore it excludes areas and animals where hunting is prohibited. Second, we conducted a case–control study that focused on positive cases of CWD, which removes spatial randomness, and certain areas were underrepresented or overrepresented depending on the occurrence of CWD. Additionally, we did not assess the age structure or relationships between harvested deer. This could have led to overrepresentation of closely related animals in the dataset due to removal through locally focused culling [46]. Also, we did not assess whether PrP variants varied by age. Finally, this dataset comes from a 20-year period of CWD surveillance in Illinois during which strain dynamics have not been assessed.

The *PRNP* amplification protocol utilized in this study avoids allelic dropout and improves the accuracy of population allele frequency estimates. The primer used and validated in this study, Ov-*PRNP*-F2, paired with the existing primer CWD-224 [22], amplified haplotypes previously unamplified due to allelic dropout. Additionally, most haplotypes previously lost to dropout encoded prion protein variant C—a variant conferring reduced CWD susceptibility—confirming that the variation resulting in allelic dropout could be co-inherited with the non-synonymous polymorphism c.286G > A (PrP G96S) [26]. However, we did not intentionally examine parents and offspring in our study. Previous work reported 18% of homozygous deer sequenced with primer pair CWD-223/CWD-224 as heterozygous when sequenced with primer pair WTDPRNP-F/WTDPRNP-R [26], designed to mitigate the effects of allelic dropout [27].

In this study, we found a higher percentage of true heterozygotes in the samples from Ishida et al. [20] (21.8%; 128/586), highlighting allelic dropout in studies utilizing primer CWD-223 [22]. While only 4.6% of the total deer sequenced with both primer sets [CWD-223/CWD-224; Ov-*PRNP*-F2/CWD-224] (128/2,736) had allelic dropout, failure to account for allelic dropout impacts allele frequency estimates for populations that have a high proportion of the c.286G > A polymorphism.

This study reports for the first time a statistically significant advantage for deer with PrP variant combination A and F over variant combination A and C. Deer expressing PrP variant combination C/F had the lowest susceptibility to CWD of the significant odds ratios (Table 2). We identified a higher proportion of CWD cases (624 PrP A/A / 778 CWD-positive deer) in animals that only encoded PrP variant A compared with animals that had at least one copy of the protective variants C (134 PrP C/[A|F] / 778 CWD-positive deer) or F (16 PrP F/[A|C] / 778 CWD-positive deer). The expanded sample size that built upon previous studies in Illinois [19–21, 32] significantly supported the finding that deer with PrP A/F are more protected than deer with PrP A/C (OR = 0.40, *p* < 0.001). Although there were not enough deer with PrP F/F to make statistically significant comparisons, there was a higher proportion of CWD cases amongst F/F deer (2/10) than C/F deer (1/88). While no conclusions can be drawn from category sizes of 2 or 1, it is unclear whether WTD expressing two different PrP variants have advantage against CWD.

Previous studies of prion infection mechanisms [3, 47–49] proved that animals with a single PrP variant expressed have a higher misfolding propensity than animals with an admixture of PrP variants. While we cannot elucidate the mechanisms of the following associations, our results may suggest that PrP variant F has a lower misfolding propensity than variant C (Figure 2, Additional file 6). The amino acid change in PrP F, Q95H, replaces a glutamine residue with histidine. The reduced misfolding propensity of PrP F can be explained by the finding that glutamine residues increase prion conversion propensity [50]. Our study considers associations between protein variant combinations and disease status, but does not disentangle the misfolding propensity of PrP variants A, C, or F. This continues to be a knowledge gap. Future work could consider utilizing protein interaction simulations such as AlphaFold [51] to investigate the relationship between protein variant expression and misfolding propensity during CWD infection.

This work adds to the body of knowledge supporting that the *PRNP* polymorphisms c.285A > C (PrP Q95H) and c.286G > A (PrP G96S) are strongly associated with the reduction of CWD in WTD. Future efforts to curb the spread of CWD could harness the population-level importance of the *PRNP* locus for herd health. One management strategy could target removing animals with only variant A (PrP A/A) while allowing animals that encode either variant C or F to reproduce. However, rapid field-based genotyping strategies of free-ranging wild animals remain undeveloped. A possible SNP detection method that could be used for field-based genotyping is loop-mediated amplification [52], to build on real-time frequency estimates of herd susceptibility to CWD. However, this is still roughly a 70-min protocol, which could limit applicability to wildlife in natural settings. A strategy targeted at removing PrP variant A animals would seemingly minimize CWD susceptibility, but may have unintended impacts, and such selection could contribute to the emergence of new strain types [53]. Furthermore, animals with PrP variant combinations including C and F could serve as silent carriers indirectly transmitting prions to the environment, which may potentiate spread. Future work is needed to assess the ideal frequencies of protective PrP variants within WTD herds.

Despite the identified advantage of variant F (PrP Q95H) over variant C (PrP G96S) in this study, some lab-based genotyping methods for determining breeding values of heritable CWD resistance do not account for the PrP Q95Hsubstitution. Based on our findings, future studies would benefit from including the *PRNP* c.285A > C polymorphism to account for the advantage conferred by PrP variant F when considering CWD risk. This would enable mapping susceptibility to CWD across the landscape by focusing on the frequencies of c.285A > C and c.286G > A polymorphisms to advance models of CWD risk and disease management strategies. Vigilance over herd genetics also could aid in tracking the emergence of prion strains capable of interspecies transmission. Prion proteins capable of misfolding propagation have been detected within wild pigs [13, 15]. Mapping the distribution of PrP variants of deer sympatric with wild pigs may also aid in tracking CWD strains and their potential to cross species barriers [51].

We have demonstrated that mitigating allelic dropout in WTD is essential for accurately estimating *PRNP* allele frequencies, especially for alleles with the advantageous polymorphism c.286G > A (G96S). Through odds ratio analysis of the largest *PRNP* sequence dataset from CWD tested wild WTD, we found a significantly greater advantage against CWD in animals expressing PrP variants C (96S) and F (95H), even when co-expressed with the high misfolding propensity variant, PrP A (95Q, 96G). This highlights the need to consider PrP variation, especially Q95H, to manage CWD in WTD herds.

## Supplementary Information

**Additional file 1** **1** **Data sources of PRNP sequences from 4076 deer**.

**Additional file 2** **Bayesian inferred PRNP haplotype frequencies of 4076 deer.**

**Additional file 3** **R Script for haplotype calling using reported PRNP sequences**.

**Additional file 4** **Translated PrP variant frequencies of 4706 deer.**

**Additional file 5** **PrP variant distribution used for analysis from 3848 CWD-tested deer.**

**Additional file 6** **PrP variant F (95H) shows the greatest advantage against CWD.**

**Additional file 7** **Distribution of PrP variant combinations used for the odds ratio calculations.**

**Additional file 8** **Median joining network (MJN) of PRNP haplotypes from 4076 Illinois white-tailed deer.**

**Additional file** **9** **PRNP haplotype distribution for 3848 CWD-tested deer**.

## Data Availability

All PRNP sequence data, translated PrP sequences, and disease test status are available in the Illinois databank; (10.13012/B2IDB-7607309_V1). For data requests, contact Evan W. London (elondon2@illinois.edu).

## References

[1] Williams ES, Young S (1980) Chronic wasting disease of captive mule deer: a spongiform encephalopathy. J Wildl Dis 16:89–98 7373730 10.7589/0090-3558-16.1.89

[2] Prusiner SB (1982) Novel proteinaceous infectious particles cause scrapie. Science 216:136–144 6801762 10.1126/science.6801762

[3] Prusiner SB (1998) Prions. Proc Natl Acad Sci U S A 95:13363–13383 9811807 10.1073/pnas.95.23.13363 PMC33918

[4] Johnson CJ, Phillips KE, Schramm PT, McKenzie D, Aiken JM, Pedersen JA (2006) Prions adhere to soil minerals and remain infectious. PLoS Pathog 2:e32 16617377 10.1371/journal.ppat.0020032 PMC1435987

[5] Basler K, Oesch B, Scott M, Westaway D, Wälchli M, Groth DF, McKinley MP, Prusiner SB, Weissman C (1986) Scrapie and cellular PrP isoforms are encoded by the same chromosomal gene. Cell 46:417–428 2873895 10.1016/0092-8674(86)90662-8

[6] Afanasieva EG, Kushnirov VV, Ter-Avanesyan MD (2011) Interspecies transmission of prions. Biochem Mosc 76:1375–1384 10.1134/S0006297911130013 22339593

[7] Kurt TD, Sigurdson CJ (2016) Cross-species transmission of CWD prions. Prion 10:83–91 26809254 10.1080/19336896.2015.1118603 PMC4981193

[8] U.S. Geological Survey. Distribution of Chronic Wasting Disease in North America Accessed 6 Jan 2026. 2025.

[9] Benestad SL, Mitchell G, Simmons M, Ytrehus B, Vikøren T (2016) First case of chronic wasting disease in Europe in a Norwegian free-ranging reindeer. Vet Res 47:88 27641251 10.1186/s13567-016-0375-4 PMC5024462

[10] Angers R, Christiansen J, Nalls AV, Kang H-E, Hunter N, Hoover E, Mathiason CK, Sheetz M, Telling GC (2014) Structural effects of PrP polymorphisms on intra- and interspecies prion transmission. Proc Natl Acad Sci U S A 111:11169–11174 25034251 10.1073/pnas.1404739111 PMC4121815

[11] Arifin MI, Hannaoui S, Chang SC, Thapa S, Schatzl HM, Gilch S (2021) Cervid prion protein polymorphisms: role in chronic wasting disease pathogenesis. Int J Mol Sci 22:2271 33668798 10.3390/ijms22052271 PMC7956812

[12] Rivera NA, Brandt AL, Novakofski JE, Mateus-Pinilla NE (2019) Chronic Wasting Disease in cervids: prevalence, impact and management strategies. Vet Med Res Rep 10:123–139 10.2147/VMRR.S197404 PMC6778748 31632898

[13] Soto P, Bravo-Risi F, Benavente R, Stimming TH, Bodenchuk MJ, Whitley P, Morales R (2025) Detection of prions in wild pigs (*Sus scrofa*) from areas with reported Chronic Wasting Disease cases, United States. Emerg Infect Dis 31:168–173 39714396 10.3201/eid3101.240401 PMC11682794

[14] Hammarström P, Nyström S (2015) Porcine prion protein amyloid. Prion 9:266–277 26218890 10.1080/19336896.2015.1065373 PMC4601310

[15] Bravo-Risi F, Brydon F, Chong A, Spicker K, Greenlee JJ, Telling G, Soto C, Pritzkow S, Barria MA, Morales R (2025) Infectious prions in brains and muscles of domestic pigs experimentally challenged with the BSE, scrapie, and CWD agents. mBio 16:e01800-25 40823831 10.1128/mbio.01800-25 PMC12421853

[16] Ott-Conn CN, Blanchong JA, Larson WA (2021) Prion protein polymorphisms in Michigan white-tailed deer (*Odocoileus virginianus*). Prion 15:183–190 34751633 10.1080/19336896.2021.1990628 PMC8583003

[17] Perrin-Stowe TIN, Ishida Y, Terrill EE, Hamlin BC, Penfold L, Cusack LM, Novakofski J, Mateus-Pinilla NE, Roca AL (2020) Prion protein gene (*PRNP*) sequences suggest differing vulnerability to Chronic Wasting Disease for Florida Key Deer (*Odocoileus virginianus clavium*) and Columbian White-Tailed Deer (*O. v. leucurus*). J Hered 111:564–572 32945850 10.1093/jhered/esaa040

[18] Robinson SJ, Samuel MD, O’Rourke KI, Johnson CJ (2012) The role of genetics in Chronic Wasting Disease of North American cervids. Prion 6:153–162 22460693 10.4161/pri.19640 PMC7082092

[19] Brandt AL, Green ML, Ishida Y, Roca AL, Novakofski J, Mateus-Pinilla NE (2018) Influence of the geographic distribution of prion protein gene sequence variation on patterns of Chronic Wasting Disease spread in white-tailed deer (*Odocoileus virginianus*). Prion 12:204–215 30041562 10.1080/19336896.2018.1474671 PMC6277178

[20] Ishida Y, Tian T, Brandt AL, Kelly AC, Shelton P, Roca AL, Novakofski J, Mateus-Pinilla NE (2020) Association of Chronic Wasting Disease susceptibility with prion protein variation in white-tailed deer (*Odocoileus virginianus*). Prion 14:214–225 32835598 10.1080/19336896.2020.1805288 PMC7518741

[21] Kelly AC, Mateus-Pinilla NE, Diffendorfer J, Jewell E, Ruiz MO, Killefer J, Shelton P, Beissel T, Novakofski J (2008) Prion sequence polymorphisms and Chronic Wasting Disease resistance in Illinois white-tailed deer (*Odocoileus virginianus*). Prion 2:28–36 19164895 10.4161/pri.2.1.6321 PMC2634418

[22] O’Rourke KI, Spraker TR, Hamburg LK, Besser TE, Brayton KA, Knowles DP (2004) Polymorphisms in the prion precursor functional gene but not the pseudogene are associated with susceptibility to Chronic Wasting Disease in white-tailed deer. J Gen Virol 85:1339–1346 15105552 10.1099/vir.0.79785-0

[23] Supattapone S, Bosque P, Muramoto T, Wille H, Aagaard C, Peretz D, Nguyen H-OB, Heinrich C, Torchia M, Safar J, Cohen FE, DeArmond SJ, Prusiner SB, Scott M (1999) Prion protein of 106 residues creates an artificial transmission barrier for prion replication in transgenic mice. Cell 96:869–878 10102274 10.1016/s0092-8674(00)80596-6

[24] Moore RA, Herzog C, Errett J, Kocisko DA, Arnold KM, Hayes SF, Priola SA (2006) Octapeptide repeat insertions increase the rate of protease-resistant prion protein formation. Protein Sci 15:609–619 16452616 10.1110/ps.051822606 PMC2249780

[25] Johnson CJ, Herbst A, Duque-Velasquez C, Vanderloo JP, Bochsler P, Chappell R, McKenzie D (2011) Prion protein polymorphisms affect chronic wasting disease progression. PLoS One 6:e17450 21445256 10.1371/journal.pone.0017450 PMC3060816

[26] Haley N, Donner R, Merrett K, Miller M, Senior K (2021) Selective breeding for disease-resistant *PRNP* variants to manage chronic wasting disease in farmed whitetail deer. Genes 12:1396 34573378 10.3390/genes12091396 PMC8471411

[27] Raudabaugh DB, Ishida Y, Haley NJ, Brown WM, Novakofski J, Roca AL, Mateus-Pinilla NE (2022) County-wide assessments of Illinois white-tailed deer (*Odocoileus virginianus*) prion protein gene variation using improved primers and potential implications for management. PLoS One 17:e0274640 36449540 10.1371/journal.pone.0274640 PMC9710747

[28] Jacques C, McDonald P. Illinois chronic wasting disease: 2023–2024 surveillance and management report. 2024.

[29] Ishida Y, Hamlin BC, Green RL, Schmidt EC, Freeman B, Gillin CM, Roca AL (2025) *PRNP* variant frequencies in Roosevelt and Rocky Mountain elk (*Cervus canadensis*) from Oregon and their implications for chronic wasting disease. J Hered 2025;esaf096 10.1093/jhered/esaf096 41247310

[30] Untergasser A, Cutcutache I, Koressaar T, Ye J, Faircloth BC, Remm M, Rozen SG (2012) Primer3–new capabilities and interfaces. Nucleic Acids Res 40:e115 22730293 10.1093/nar/gks596 PMC3424584

[31] Edgar RC (2022) Muscle5: high-accuracy alignment ensembles enable unbiased assessments of sequence homology and phylogeny. Nat Commun 13:6968 36379955 10.1038/s41467-022-34630-w PMC9664440

[32] Brandt AL, Kelly AC, Green ML, Shelton P, Novakofski J, Mateus-Pinilla NE (2015) Prion protein gene sequence and chronic wasting disease susceptibility in white-tailed deer (*Odocoileus virginianus*). Prion 9:449–462 26634768 10.1080/19336896.2015.1115179 PMC4964855

[33] Stephens M, Smith NJ, Donnelly P (2001) A new statistical method for haplotype reconstruction from population data. Am J Hum Genet 68:978–989 11254454 10.1086/319501 PMC1275651

[34] Stephens M, Donnelly P (2003) A comparison of Bayesian methods for haplotype reconstruction from population genotype data. Am J Hum Genet 73:1162–1169 14574645 10.1086/379378 PMC1180495

[35] Rozas J, Ferrer-Mata A, Sánchez-DelBarrio JC, Guirao-Rico S, Librado P, Ramos-Onsins SE, Sánchez-Garcia A (2017) DnaSP 6: DNA sequence polymorphism analysis of large data sets. Mol Biol Evol 34:3299–3302 29029172 10.1093/molbev/msx248

[36] Tamura K, Stecher G, Kumar S (2021) MEGA11: molecular evolutionary genetics analysis version 11. Mol Biol Evol 38:3022–3027 33892491 10.1093/molbev/msab120 PMC8233496

[37] Hahsler M, Annurag N. rBLAST: R Interface for the basic local alignment search tool. Bioconductor version: release (3.19). R package version 0.99.4. 2024.

[38] Camacho C, Coulouris G, Avagyan V, Ma N, Papadopoulos J, Bealer K, Madden TL (2009) BLAST+: architecture and applications. BMC Bioinformatics 10:421 20003500 10.1186/1471-2105-10-421 PMC2803857

[39] Fisher RA (1935) The logic of inductive inference. J R Stat Soc 98:39–82

[40] Benjamini Y, Hochberg Y (1995) Controlling the false discovery rate: a practical and powerful approach to multiple testing. J R Stat Soc B Methodol 57:289–300

[41] Benjamini Y, Yekutieli D (2001) The control of the false discovery rate in multiple testing under dependency. Ann Stat 29:1165–1188

[42] Chafin TK, Douglas MR, Martin BT, Zbinden ZD, Middaugh CR, Ballard JR, Gray MC, White D Jr, Douglas ME (2020) Age structuring and spatial heterogeneity in prion protein gene (*PRNP*) polymorphism in white-tailed deer. Prion 14:238–248 33078661 10.1080/19336896.2020.1832947 PMC7575228

[43] Bandelt HJ, Forster P, Röhl A (1999) Median-joining networks for inferring intraspecific phylogenies. Mol Biol Evol 16:37–48 10331250 10.1093/oxfordjournals.molbev.a026036

[44] Johnson C, Johnson J, Vanderloo JP, Keane D, Aiken JM, McKenzie D (2006) Prion protein polymorphisms in white-tailed deer influence susceptibility to chronic wasting disease. J Gen Virol 87:2109–2114 16760415 10.1099/vir.0.81615-0

[45] Johnson C, Johnson J, Clayton M, McKenzie D, Aiken J (2003) Prion protein gene heterogeneity in free-ranging white-tailed deer within the chronic wasting disease affected region of Wisconsin. J Wildl Dis 39:576–581 14567218 10.7589/0090-3558-39.3.576

[46] Fameli A, Jennelle C, Edson J, Hildebrand E, Carstensen M, Walter WD (2025) Relatedness of white-tailed deer from culling efforts within chronic wasting disease management zones in Minnesota. Pathogens 14:67 39861028 10.3390/pathogens14010067 PMC11768294

[47] Caughey B (2003) Prion protein conversions: insight into mechanisms, TSE transmission barriers and strains. Br Med Bull 66:109–120 14522853 10.1093/bmb/66.1.109

[48] Hizume M, Kobayashi A, Teruya K, Ohashi H, Ironside JW, Mohri S, Kitamoto T (2009) Human Prion Protein (PrP) 219K is converted to PrPSc but shows heterozygous inhibition in variant Creutzfeldt-Jakob disease infection. J Biol Chem 284:3603–3609 19074151 10.1074/jbc.M809254200

[49] Kobayashi A, Hizume M, Teruya K, Mohri S, Kitamoto T (2009) Heterozygous inhibition in prion infection. Prion 3:27–30 19372732 10.4161/pri.3.1.8514 PMC2676740

[50] Kurt TD, Aguilar-Calvo P, Jiang L, Rodriguez JA, Alderson N, Eisenberg DS, Sigurdson CJ (2017) Asparagine and glutamine ladders promote cross-species prion conversion. J Biol Chem 292:19076–19086 28931606 10.1074/jbc.M117.794107 PMC5704488

[51] Abramson J, Adler J, Dunger J, Evans R, Green T, Pritzel A, Ronneberger O, Willmore L, Ballard AJ, Bambrick J, Bodenstein SW, Evans DA, Hung C-C, O’Neill M, Reiman D, Tunyasuvunakool K, Wu Z, Žemgulytė A, Arvaniti E, Beattie C, Bertolli O, Bridgland A, Cherepanov A, Congreve M, Cowen-Rivers AI, Cowie A, Figurnov M, Fuchs FB, Gladman H, Jain R, Khan YA et al (2024) Accurate structure prediction of biomolecular interactions with AlphaFold 3. Nature 630:493–500 38718835 10.1038/s41586-024-07487-w PMC11168924

[52] Hyman LB, Christopher CR, Romero PA (2022) Competitive SNP-LAMP probes for rapid and robust single-nucleotide polymorphism detection. Cell Rep Methods 2:100242 35880021 10.1016/j.crmeth.2022.100242 PMC9308130

[53] Otero A, Duque Velasquez C, McKenzie D, Aiken J (2023) Emergence of CWD strains. Cell Tissue Res 392:135–148 36201049 10.1007/s00441-022-03688-9 PMC10113326

