# Supplementary material for: Odocoileus virginianus PRNP sequencing reveals AF (Q95G96/H95G96) advantage over AC (Q95G96/Q95S96) against chronic wasting disease
Source: Vet Res. 2026 May 26;57:84. doi: 10.1186/s13567-026-01752-8 (PMC13214280; doi:10.1186/s13567-026-01752-8)
Supplement: Supplementary file 1 — Additional file 1 1 Data sources of PRNP sequences from 4076 deer. [file 13567_2026_1752_MOESM1_ESM.pdf]

Data source [Reference #]	# of Deer	CWD-Status *Negative*	*Positive*	*Origin/Status Unknown*	Year Range	***PRNP*** **PCR Primers (Forward/Reverse)**	# PrP Codons Read
Kelly et al. 2008, Brandt et al. 2015 & 2018† [19, 21, 32]	1838	1446	165	227	2002-2015	CWD-223/CWD-224	207
Ishida et al. 2020† [20]	312	203	108	1	2015-2017	CWD-223/CWD-224	257
Homozygote Resequencing*	586	495	91	0	2002-2017	Ov-*PRNP* F2/CWD-224	208
Total Previous Samples	2736	2144	364	228	2002-2017	--	--
Present Study	1340	926	414	0	2018-2022	Ov-*PRNP* F2/CWD-224	208
Sample set for Bayesian Calling	4076	3070	778	228	2002-2022	--	208
Sample set for Analysis	3848	3070	778	0	2002-2022	--	208

†Previous sample numbers are greater than those used in this study due to homozygous resequencing and available tissue.

*Resequencing of available homozygous samples from Kelly et al. 2008, Brandt et al. 2015 & 2018, and Ishida et al. 2020 [19-21, 32]
