# Supplementary material for: Odocoileus virginianus PRNP sequencing reveals AF (Q95G96/H95G96) advantage over AC (Q95G96/Q95S96) against chronic wasting disease
Source: Vet Res. 2026 May 26;57:84. doi: 10.1186/s13567-026-01752-8 (PMC13214280; doi:10.1186/s13567-026-01752-8)
Supplement: Supplementary file 2 — Additional file 2 Bayesian inferred PRNP haplotype frequencies of 4076 deer. [file 13567_2026_1752_MOESM2_ESM.pdf]

Additional File 2 – Bayesian inferred *PRNP* haplotype frequencies of 4,076 deer.

Haplotype	Nucleotide position within the *PRNP* coding region (PrP amino acid residue)																Number of chromosomes	Freq.
	60 (20)	153 (51)	243 (81)	**285 (95)**	**286 (96)**	**299 (100)**	**308 (103)**	324 (108)	**367 (123)**	372 (124)	378 (126)	438 (146)	441 (147)	499 (167)	555 (185)	**676 (226)**		
A	C	C	T	A	G	G	A	A	G	G	G	C	C	A	C	C	2372	0.2910
B	.	.	.	.	.	.	.	.	.	.	.	.	.	.	T	.	1980	0.2429
C	.	.	.	.	A	.	.	.	.	.	.	.	.	.	T	.	1458	0.1789
D	.	T	.	.	.	.	.	.	.	.	.	.	.	.	.	.	895	0.1098
E	.	.	.	.	.	.	.	.	.	.	.	T	.	.	.	.	350	0.0429
F	T	.	.	C	.	.	.	.	.	.	.	.	.	.	.	.	383	0.0470
G	T	.	.	.	.	.	.	.	.	.	.	.	.	.	.	.	292	0.0358
H	.	.	.	.	.	.	.	.	.	A	.	.	.	.	T	.	85	0.0104
I	.	.	A	.	A	.	.	.	.	.	.	.	.	.	T	.	63	0.0077
J	.	.	.	.	.	.	.	G	.	.	.	.	.	.	.	.	77	0.0094
K	T	.	.	.	.	.	.	.	.	.	.	.	.	.	.	A	34	0.0042
L	.	.	.	.	.	.	.	.	A	.	.	.	.	.	.	.	39	0.0048
M	.	.	.	.	.	A	.	.	.	.	.	.	.	.	.	.	4	0.0005
N	T	.	.	C	A	.	.	.	.	.	.	.	.	.	.	.	7	0.0009
O	T	T	.	.	.	.	.	.	.	.	.	.	.	.	.	.	29	0.0036
OVC1/AR2	.	.	.	.	A	.	.	.	.	.	.	.	.	C	T	.	1	0.0001
PRNP-Odvi27	.	.	A	.	A	.	.	.	.	.	.	.	.	.	.	.	2	0.0002
PRNP-Odvi28	.	.	.	.	A	.	.	.	.	.	.	T	.	.	T	.	3	0.0004
PRNP-Odvi29	.	.	.	.	C	.	.	.	.	.	.	.	.	.	T	.	1	0.0001
PRNP-Odvi30	.	.	.	.	C	.	.	G	.	.	.	.	.	.	.	.	1	0.0001
PRNP-Odvi31	.	.	.	.	.	.	.	.	.	.	.	T	.	.	T	.	1	0.0001
PRNP-Odvi32	.	T	.	.	.	.	.	.	.	.	.	.	T	.	.	.	1	0.0001
PRNP-Odvi33	.	T	.	.	.	.	.	.	.	.	.	T	.	.	.	.	2	0.0002
PRNP-Odvi34	T	.	.	.	A	.	.	.	.	.	.	.	.	.	T	.	2	0.0002
PRNP-Odvi35	T	.	.	C	.	.	.	.	.	.	.	.	T	.	.	.	1	0.0001
PRNP-Odvi36	T	.	.	G	.	.	.	.	.	.	.	.	.	.	.	.	5	0.0006
PRNP-Odvi37	T	T	.	C	.	.	.	.	.	.	.	.	.	.	.	.	3	0.0004
PRNP-Odvi38	T	.	.	.	.	.	.	.	.	.	.	.	.	.	T	.	1	0.0001
P	.	.	.	.	A	.	.	.	.	.	.	.	.	.	.	.	12	0.0015
Q	.	.	.	.	A	.	.	.	A	.	.	.	.	.	T	.	2	0.0002
R	.	T	.	.	.	.	.	.	.	.	.	.	.	.	T	.	12	0.0015
S		.	.	.	A	A	.	.	.	.	.	.	.	.	.	.	1	0.0001
T	.	T	.	.	.	.	.	.	.	.	A	.	.	.	.	.	4	0.0005
U	.	T	.	.	.	.	T	.	.	.	.	.	.	.	.	.	19	0.0023
V	.	T	.	.	A	.	.	.	.	.	.	.	.	.	.	.	4	0.0005
W	T	.	.	.	A	.	.	.	.	.	.	.	.	.	.	.	2	0.0002
X	T	.	A	.	A	.	.	.	.	.	.	.	.	.	.	.	2	0.0002
Y	.	.	.	C	.	.	.	.	.	.	.	.	.	.	.	.	2	0.0002
