# Supplementary material for: Odocoileus virginianus PRNP sequencing reveals AF (Q95G96/H95G96) advantage over AC (Q95G96/Q95S96) against chronic wasting disease
Source: Vet Res. 2026 May 26;57:84. doi: 10.1186/s13567-026-01752-8 (PMC13214280; doi:10.1186/s13567-026-01752-8)
Supplement: Supplementary file 3 — Additional file 3 R Script for haplotype calling using reported PRNP sequences. [file 13567_2026_1752_MOESM3_ESM.pdf]

Additional File 3 – R Script for haplotype calling using reported *PRNP* sequences.

```
library(rBLAST)

# Create an R object for the DNA database
dna_db <- blast(db="../DNA_Directory/db_prefix")
# Create an R object for the Protein database
prot_db <- blast(db="../Protein_Directory/db_prefix", type = "blastp")

# Load in the phased nucleotide sequences generated by DNASP6
phased_dna <- sort(readDNAStringSet("./phased_DNA_DNASP6.fas"))
# Load in the phased protein sequences translated by MEGA
phased_protein <- sort(readAAStringSet("./phased_Protein_MEGA.fas"))

# Use nucleotide blast to query the nucleotide database with the phased DNA sequences
dna_result <- predict(dna_db, phased_dna, BLAST_args = "-max_target_seqs 1 -max_hsps 1")
# Use protein blast to query the protein database with the phased protein sequences
protein_result <- predict(prot_db, phased_protein, BLAST_args = "-max_target_seqs 1 -max_hsps 1")

# Subset any data that needs to be error-proofed (does not match back to databases)
dna_error <- dna_result[which(dna_result$pident<100),]
prot_error <- protein_result[which(protein_result$pident<100),]
```
