# Supplementary material for: Odocoileus virginianus PRNP sequencing reveals AF (Q95G96/H95G96) advantage over AC (Q95G96/Q95S96) against chronic wasting disease
Source: Vet Res. 2026 May 26;57:84. doi: 10.1186/s13567-026-01752-8 (PMC13214280; doi:10.1186/s13567-026-01752-8)
Supplement: Supplementary file 4 — Additional file 4 Translated PrP variant frequencies of 4706 deer. [file 13567_2026_1752_MOESM4_ESM.pdf]

Additional File 4 – Translated PrP variant frequencies of 4,706 deer.

		PRNP nucleotide position							
		285	286	299	308	367	676		
		PrP amino acid residue							
PrP Protein Variant	Encoding *PRNP* haplotypes	95	96	100	103	123	226	Number of Chromosomes	Frequency
A	A, B, D, E, F, G, H, J, O, Odvi31, Odvi32, Odvi33, Odvi34, Odvi36, Odvi38	Q	G	S	N	A	Q	6106	0.7490
C	C, I, OVC1, Odvi27, Odvi28, Odvi38, P, V, W, X	.	S	.	.	.	.	1549	0.1900
F	F, Y, Odvi35, Odvi37	H	.	.	.	.	.	389	0.0477
K	K	.	.	.	.	.	K	34	0.0042
L	L	.	.	.	.	T	.	39	0.0048
M	M			N				4	0.0005
N	N	H	S	.	.	.	.	7	0.0009
Odvi29	Odvi29, Odvi30	.	R	.	.	.	.	2	0.0002
Q	Q	.	S	.	.	T	.	2	0.0002
S	S		S	N				1	0.0001
U	U	.	.	.	I	.	.	19	0.0023
