# Supplementary material for: Odocoileus virginianus PRNP sequencing reveals AF (Q95G96/H95G96) advantage over AC (Q95G96/Q95S96) against chronic wasting disease
Source: Vet Res. 2026 May 26;57:84. doi: 10.1186/s13567-026-01752-8 (PMC13214280; doi:10.1186/s13567-026-01752-8)
Supplement: Supplementary file 5 — Additional file 5 PrP variant distribution used for analysis from 3848 CWD-tested deer. [file 13567_2026_1752_MOESM5_ESM.pdf]

Additional File 5 – PrP variant distribution used for analysis from 3,848 CWD-tested deer.

		PRNP nucleotide position								
		285	286	308	367	676				
		PrP amino acid residue					Number of Chromosomes			
PrP Protein Variant	Encoding *PRNP* haplotypes	95	96	103	123	226	CWD (-)	CWD (+)	Total	Frequency
A	A, B, D, E, F, G, H, J, O, Odvi31, Odvi32, Odvi33, Odvi34, Odvi36, Odvi38	Q	G	N	A	Q	4376	1387	5763	0.7488
C	C, I, OVC1, Odvi27, Odvi28, Odvi38, P, V, W, X	.	S	.	.	.	1322	146	1468	0.1907
F	F, Y, Odvi35, Odvi37	H	.	.	.	.	349	18	367	0.0477
K	K	.	.	.	.	K	29	3	32	0.0042
L	L	.	.	.	T	.	37	2	39	0.0051
N	N	H	S	.	.	.	5	0	5	0.0006
Odvi29	Odvi29, Odvi30	.	R	.	.	.	2	0	2	0.0003
Q	Q	.	S	.	T	.	2	0	2	0.0003
U	U	.	.	I	.	.	18	0	18	0.0023
