# Supplementary material for: Odocoileus virginianus PRNP sequencing reveals AF (Q95G96/H95G96) advantage over AC (Q95G96/Q95S96) against chronic wasting disease
Source: Vet Res. 2026 May 26;57:84. doi: 10.1186/s13567-026-01752-8 (PMC13214280; doi:10.1186/s13567-026-01752-8)
Supplement: Supplementary file 6 — Additional file 6 PrP variant F (95H) shows the greatest advantage against CWD. [file 13567_2026_1752_MOESM6_ESM.pdf]

Additional File 6 – PrP variant F (95H) shows the greatest advantage against CWD.

Group 1 vs. Group 2	Odds Ratio	95% Confidence Interval	Group 1		Group 2	
			CWD -	CWD +	CWD -	CWD +
A vs. C	0.34*	0.28 - 0.41	4376	1387	1322	146
A vs. F	0.16*	0.09 - 0.26	4376	1387	349	18
C vs. F	0.46†	0.26 - 0.77	1322	146	349	18
C vs. A	2.86*	2.38 - 3.46	1322	146	4367	1387
F vs. A	6.13*	3.80 – 10.52	349	18	4367	1387
F vs. C	2.14†	1.28 – 3.76	349	18	1322	146

*Significant at $p < 0.001$, †significant at $p < 0.01$

Underlying calculations for odds ratios of deer testing positive for CWD based upon the number chromosomes encoding PrP variants presented in Figure 2. PrP variants with greater than 1% population frequency (A, C, and F) within Illinois white-tailed deer were used for odds ratio comparisons. The number of chromosomes in CWD-positive and CWD-negative deer within each comparison group are noted. Reciprocal protein variant odds ratios (below black line) are shown for comparison purposes and to further demonstrate CWD vulnerability conferred by each variant.
