# Supplementary material for: Odocoileus virginianus PRNP sequencing reveals AF (Q95G96/H95G96) advantage over AC (Q95G96/Q95S96) against chronic wasting disease
Source: Vet Res. 2026 May 26;57:84. doi: 10.1186/s13567-026-01752-8 (PMC13214280; doi:10.1186/s13567-026-01752-8)
Supplement: Supplementary file 7 — Additional file 7 Distribution of PrP variant combinations used for the odds ratio calculations. [file 13567_2026_1752_MOESM7_ESM.pdf]

Additional File 7 – Distribution of PrP variant combinations used for the odds ratio calculations.

PrP variant combination (Animal-level)	CWD-negative Deer	CWD-positive Deer
A/A	1588	624
A/C	908	122
A/F	241	13
C/C	152	11
C/F	87	1
F/F	8	2

The number of animals testing negative and positive by protein variant combination with PrP A, C, and F. Animals with PrP variants present at less than 1% population frequency were excluded from odds ratio comparison groups.
