# Supplementary material for: Odocoileus virginianus PRNP sequencing reveals AF (Q95G96/H95G96) advantage over AC (Q95G96/Q95S96) against chronic wasting disease
Source: Vet Res. 2026 May 26;57:84. doi: 10.1186/s13567-026-01752-8 (PMC13214280; doi:10.1186/s13567-026-01752-8)
Supplement: Supplementary file 8 — Additional file 8 Median joining network (MJN) of PRNP haplotypes from 4076 Illinois white-tailed deer. [file 13567_2026_1752_MOESM8_ESM.pdf]

Additional File 8 - Median joining network (MJN) of *PRNP* haplotypes from 4,076 Illinois white-tailed deer.

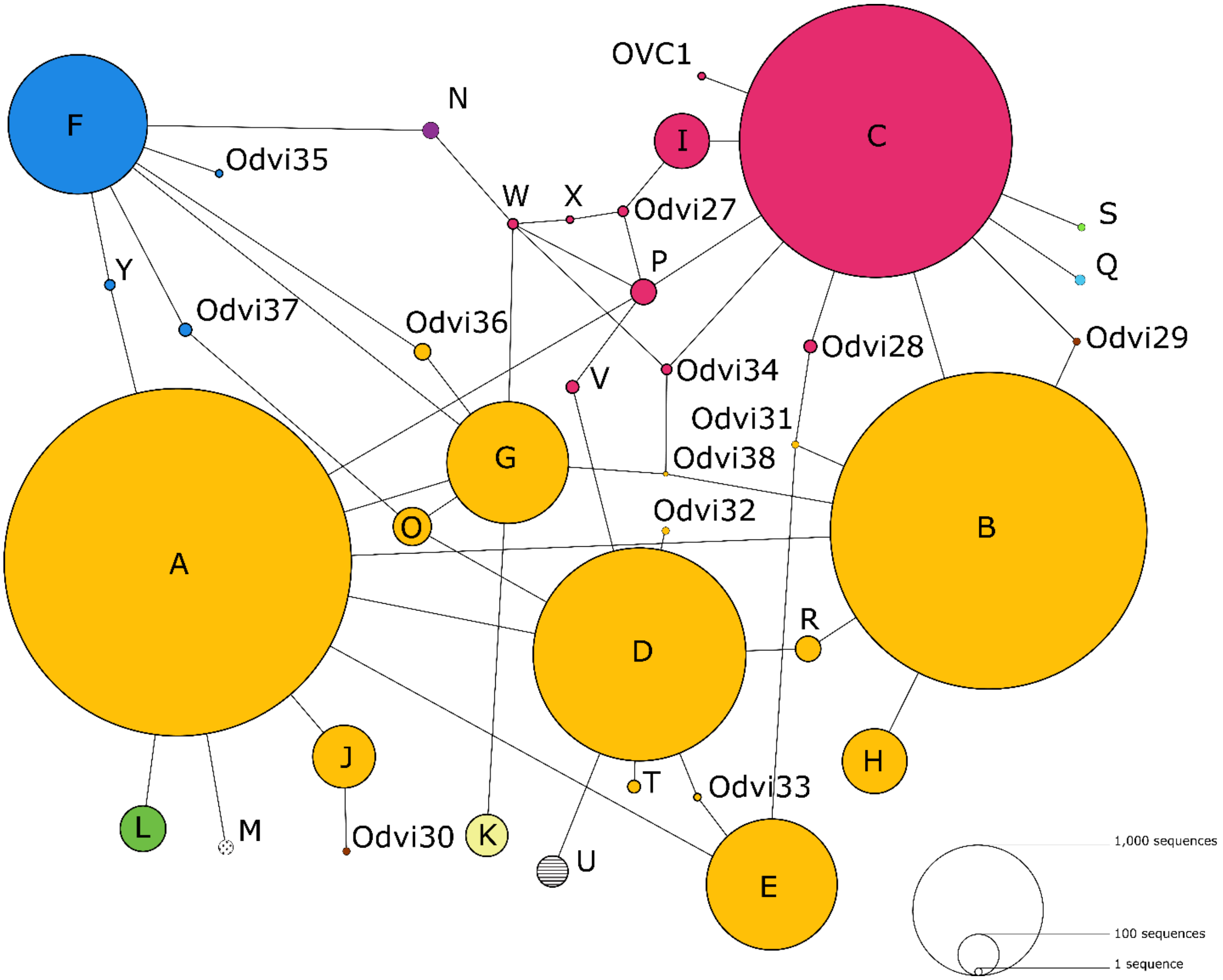

Circles in the network represent haplotypes and the color corresponds to the prion protein (PrP) variant encoded by the haplotype. Lines represent one-step nucleotide changes between haplotypes. No median haplotypes were inferred in the construction of the MJN. Only PrP Odvi29 had haplotypes separated by two nucleotide changes (Odvi29, Odvi30). Visual grouping of haplotypes is based on the encoded PrP variant, with variants <0.5% population frequency (K,L,M,N,Odvi29,) along the outer edges of the network and ensuring that no lines were obscured.
